# The DyP-type peroxidase DtpA is a Tat-substrate required for GlxA maturation and morphogenesis in *Streptomyces*

**DOI:** 10.1098/rsob.150149

**Published:** 2016-01-06

**Authors:** Marloes L. C. Petrus, Erik Vijgenboom, Amanda K. Chaplin, Jonathan A. R. Worrall, Gilles P. van Wezel, Dennis Claessen

**Affiliations:** 1 Molecular Biotechnology, Institute of Biology, Leiden University, Sylviusweg 72, 2333 BE Leiden, The Netherlands; 2 School of Biological Science, University of Essex, Wivenhoe Park, Colchester CO4 3SQ, UK

**Keywords:** morphogenesis, twin-arginine translocation, DyP-type peroxidase, cuproenzyme, GlxA maturation, glycan

## Abstract

The filamentous bacterium *Streptomyces lividans* depends on the radical copper oxidase GlxA for the formation of reproductive aerial structures and, in liquid environments, for the formation of pellets. Incorporation of copper into the active site is essential for the formation of a cross-linked tyrosyl-cysteine cofactor, which is needed for enzymatic activity. In this study, we show a crucial link between GlxA maturation and a group of copper-related proteins including the chaperone Sco and a novel DyP-type peroxidase hereinafter called DtpA. Under copper-limiting conditions, the *sco* and *dtpA* deletion mutants are blocked in aerial growth and pellet formation, similarly to a *glxA* mutant. Western blot analysis showed that GlxA maturation is perturbed in the *sco* and *dtpA* mutants, but both maturation and morphology can by rescued by increasing the bioavailability of copper. DtpA acts as a peroxidase in the presence of GlxA and is a substrate for the twin-arginine translocation (Tat) translocation pathway. In agreement, the maturation status of GlxA is also perturbed in *tat* mutants, which can be compensated for by the addition of copper, thereby partially restoring their morphological defects. Our data support a model wherein a copper-trafficking pathway and Tat-dependent secretion of DtpA link to the GlxA-dependent morphogenesis pathway.

## 1. Introduction

Streptomycetes are multicellular bacteria with a complex developmental life cycle. Following the germination of spores, a network of interconnected filaments is established, which is called a vegetative mycelium. This mycelium feeds on nutrients in the soil until they become depleted. This nutrient scarcity triggers the onset of a developmental programme, leading to the lysis of the vegetative mycelium, and the formation of aerial hyphae that erect from the colony surface into the air, and which gives the colony a white, fluffy appearance [1,2]. Differentiation of these reproductive structures leads to the synchronous production of millions of grey-pigmented spores that easily disperse. At the onset of aerial mycelium formation, streptomycetes produce a richness of secondary metabolites, including numerous antibiotics, anti-tumour compounds and anthelmintic agents that make them of interest for pharmaceutical purposes [3,4]. Additionally, owing to their competence to directly secrete proteins in the culture broth, streptomycetes hold promise as hosts for the heterologous production of enzymes [5,6].

Metabolite and enzyme production typically occurs in large bioreactors. Growth under these conditions is characterized by the formation of large, biofilm-like aggregates of mycelium, called pellets [7,8]. Like in biofilms, formation and integrity of these structures depends on the synthesis of extracellular glycans [9–11]. Recently, reverse engineering of a non-pelleting strain of *Streptomyces lividans* indicated a crucial role for the newly identified *mat* gene locus, putatively involved in synthesis of an extracellular glycan needed for pellet formation [12]. Deletion of the *mat* genes leads to a dispersed mycelium with a 60% increase in growth rate and productivity of *S. lividans* [12]. A second extracellular glycan involved in pellet formation is produced by enzymes encoded by the *cslA–glxA* locus [13–15]. The *cslA* gene encodes a protein belonging to family 2 of the glycosyl transferases, which contains cellulose and chitin synthases, among others [16]. CslA synthesizes a *β*-(1,4)-glycan at hyphal tips, which is thought to provide protection during the ongoing cell wall remodelling at these sites [15]. Mutation of *cslA* not only abolishes pellet formation in liquid-grown cultures, but also blocks aerial growth [13,15]. The *cslA* gene is located in an operon with the downstream located *glxA* gene. The *cslA–glxA* operon is probably acquired via horizontal gene transfer and is conserved among all streptomycetes, with some species having two copies [17]. In most streptomycetes, this gene cluster also contains a third gene downstream of *glxA*, called *cslZ*, which encodes an endoglucanase [15,17]. Like in the absence of *cslA*, deletion of *glxA* blocks development and abolishes pellet formation, coinciding with the loss of glycan deposition at hyphal tips [14,17]. This is consistent with a model in which both proteins cooperatively function in glycan deposition.

GlxA has been recently characterized [14]. The X-ray crystal structure revealed a unique tertiary structure with an active site consisting of a mononuclear copper (Cu) ion and a tyrosyl-cysteine redox cofactor, bearing resemblance to the Cu active site in fungal galactose oxidases (Gox) [18]. Enzymes of this family carry out the two-electron oxidation of primary alcohols to aldehydes with the reduction of dioxygen to hydrogen peroxide [19]. Unlike Gox, the active site Cu and putative substrate binding pocket is buried in GlxA, but can be accessed through channels leading down from three separate surface locations [14]. Notably, no significant *in vitro* enzymatic activity with d-galactose or a range of mono- or disaccharide substrates that are turned over by Gox was detected [14,20]. However, GlxA was able to turnover glycolaldehyde, the smallest molecule to contain both an aldehyde and a hydroxyl group. Thus, it is likely that the substrate specificity of GlxA is different from that of Gox.

*Streptomyces lividans* strongly depends on Cu to initiate the morphological switch from vegetative to aerial growth [21,22]. Our previous work provided clues for the existence of a Cu-trafficking pathway involved in this process. One of the proteins in this pathway, the Cu chaperone Sco, is required for morphogenesis under conditions of low Cu availability. Notably, morphogenesis of the *sco* mutant is restored by the addition of Cu to the medium [23]. Sco receives its Cu ion from the extracytoplasmic Cu chaperone ECuC [24], and, in turn, delivers Cu to the Cu_A_ site of an *aa_3_*-type cytochrome *c* oxidase (CcO) and to a second target, possibly the cuproenzyme GlxA, that is required to trigger aerial growth [14,23,24]. In contrast to the *sco* mutant, the *glxA* phenotype (on solid media or in solution) cannot be rescued by the addition of exogenous Cu [14].

*sco* (SLI_4214) and *ecuc* (SLI_4213) are the first two genes of an operon that also contains genes for a putative Cu transport protein (SLI_4212) and for a secreted protein with a putative twin-arginine translocation (Tat) signal sequence (SLI_4211) [25] and a dye-decolourizing peroxidase (DyP)-type domain [23,26,27]. DyPs are a new class of monohaem peroxidases that are widely distributed among bacteria and fungi, but their physiological role remains unclear [28,29]. Here, we show that SLI_4211, hereinafter called *dtpA* (for DyP-type peroxidase A), encodes a protein that functions as a peroxidase in the presence of GlxA and is required for executing a crucial enzymatic step in the cascade of the GlxA-dependent morphogenesis pathway. Deletion of the *dtpA* gene leads to an arrest in development owing to impaired GlxA maturation and function, which can be overcome by the extracellular addition of Cu to the medium. Extracellular complementation with Cu also restores GlxA maturation issues and development in *tat* mutants, thereby connecting Tat-dependent secretion of DtpA to GlxA-dependent morphogenesis. We propose an integrated model for how a Cu-trafficking pathway and Tat secretion ultimately link to the GlxA-dependent morphogenesis pathway.

## 2. Methods

### 2.1. Bacterial strains and plasmids

All *Streptomyces* strains used in this study are presented in table 1. Mutants were constructed in *S. lividans* 1326 (*S. lividans* 66, stock number 1326 from the John Innes Centre) [30]. The *tat* mutants, kindly provided by Dr J. Anné and Dr L. Vanmellaert (Katholieke Universiteit Leuven), were created in the *S. lividans* TK24 background [32–34]. *Escherichia coli* JM109 was used for routine cloning purposes [35]. Vectors and constructs are summarized in table 2.

**Table 1.** *Streptomyces lividans* strains used in this study.

strains	description	reference or source
1326	wild-type *S. lividans* 1326	[30]
Δ*cslA*	1326 lacking *cslA* (marker-less)	[14]
Δ*glxA*	1326 lacking *glxA* (marker-less)	[14]
Δ*cslZ*	1326 lacking *cslZ* (marker-less)	this work
Δ*sco*	1326 lacking *sco* (marker-less)	[23]
Δ*ecuc*	1326 lacking *ecuc* (marker-less)	[24]
Δ*dtpA*	1326 lacking *dtpA* (marker-less)	this work
ΔSLI_4212	1326 lacking SLI_4212 (marker-less)	this work
Δ*cox*	1326 SLI_2481-2482::*aac(3)IV*	[23]
TK24	*S. lividans* TK24	[31]
Δ*tatA*	TK24 *tatA::aac(3)IV*	[32]
Δ*tatB*	TK24 *tatB::aac(3)IV*	[33]
Δ*tatC*	TK24 *tatC::neo*	[34]

**Table 2.** Vectors and constructs used in this study.

plasmid	description	reference
pSET152	*Streptomyces/E. coli* shuttle vector. Integrates in *Streptomyces*	[36]
pWHM3	*Streptomyces/E. coli* shuttle vector	[37]
pΔ*cslZ*	pWHM3 derivative containing the flanking regions of the *S. lividans cslZ gene* (SLI_3189) interspersed by the apra-*loxP* cassette	this work
pΔ*dtpA*	pWHM3 derivative containing the flanking regions of the *S. lividans dtpA* gene (SLI_4211) interspersed by the apra-*loxP* cassette	this work
pΔSLI_4212	pWHM3 derivative containing the flanking regions of the *S. lividans* SLI_4212 gene interspersed by the apra-*loxP* cassette	this work
pTDW46	pSET152 derivative containing the *dagA* gene, where the sequence corresponding to the original DagA signal peptide is replaced by *aadA*, the streptomycin resistance gene	[25]
pTDW47	pTDW46 containing a fragment encoding the DagA signal peptide	[25]
pMLCP1	pSET152 derivative with *dtpA* under control of the *sco* promoter	this work
pMLCP2	pTDW46 derivative containing a fragment encoding the putative DtpA signal sequence (MPDQSIPQTRSPEATRGTPGPLDSDNPGAATAPEGVSRRRLLGTAGATGLVLGAAGAAAGYAAAPSSAATPLTSLGSGS)	this work
pMLCP3	pTDW46 derivative containing a fragment encoding the CslZ signal sequence (MYGSKPAGNMSRRRAASAAALGAAALLLAGCSSSGDGDDKAAGAGITQQPKETDGS)	this work
pMLCP4	pTDW46 derivative containing a fragment encoding the putative ECuC signal sequence (MRRLAEGPVRRLAGGPVRRRPALAAVAVIGALTLAGCGGSDSGADSASPGAELSVDAAGS)	this work
pMLCP5	pTDW46 derivative containing a fragment encoding the putative GlxA signal sequence (MKDRAGRRRARRFAIGTAVVVALAGMNGPWLGS)	this work
pET4211	pET28a vector with N-terminal His-tag, containing the *dtpA* (SLI_4211) gene restricted between the *Nde*I and *Hind*III sites	this work
pET3188	pET28a vector with N-terminal His-tag, containing the *glxA* (SLI_3188) gene encoding residues 35-645, restricted between the *Nde*I and *Hind*III sites	[14]

### 2.2. Growth conditions and media

*Streptomyces* strains were grown at 30°C [31]. *Streptomyces* spores were isolated from soy flour-mannitol (MS) agar plates [38]. For phenotypical characterizations, ±10^6^ spores were plated in square 2 × 2 cm patches on R5 agar plates, supplemented with 10 µM CuSO_4,_ FeSO_4_, MnSO_4_, ZnSO_4_ or Co(NO_3_)_2_ if necessary. Photographs of plates were taken daily with a compact digital camera (Canon Ixus).

For morphology in liquid-grown cultures, tryptic soy broth with 10% sucrose (TSBS) was used, which was supplemented with 10 µM CuSO_4_ as indicated; 250 ml flasks equipped with coils and containing 100 ml TSBS medium were inoculated with 10^6^ spores ml^−1^. Morphology was determined following 24 h growth at 30°C while shaking at 200 r.p.m. Samples from liquid-grown cultures were analysed by light microscopy with a Zeiss standard 25 microscope and digital pictures were taken with an AxioCam linked to AxioVision software.

### 2.3. Construction of the *cslZ*, *dtpA* and SLI_4212 mutants

The *cslZ, dtpA* and SLI_4212 null mutants were created in *S. lividans* 1326 in a two-step process using the unstable pWHM3 plasmid and the Cre-LoxP system as described [39]. In the *cslZ* null mutant, nucleotides +15 to +1011 relative to the start codon of SLI_3188 were replaced with the *loxP-apra* cassette, whereas in the *dtpA* mutant nucleotides +19 to +1208 relative to the start of SLI_4211 and in the SLI_4212 mutant nucleotides −18 to +1932 relative to the start of SLI_4212 were replaced. The Cre recombinase was used to remove the *loxP-apra* cassette from the obtained mutants, which were then verified by PCR amplification and sequencing. The *dtpA* mutant was complemented by integration of plasmid pMLCP1 (table 2), which contains the *dtpA* gene under the control of the *sco* promoter. All primers used in this work are shown in table 3.

**Table 3.** Primers used in this study. The used restriction sites are highlighted in italics.

primer name	primer sequence	restriction site
SLI_4211.P1	GATC*GAATTC*CTGCTGCGCGGCTCGTACAC	*Eco*RI
SLI_4211.P2	CATG*TCTAGA*AATGGACTGGTCGGGCATGG	*Xba*I
SLI_4211.P3	GATC*TCTAGA*CGTGCAGCGCAAGCTGGACC	*Xba*I
SLI_4211.P4	CATG*AAGCTT*ATGAAGCGCTGCGGAATCCC	*Hind*III
4211.FW.NdeI	GATC*CATATG*CCCGACCAGTCCATTCC	*Nde*I
4211.RV.XbaI	CATG*TCTAGA*GCCTTCAGGGCCGAGATACG	*Xba*I
4211-F	CTAA*CATATG*GCGACTCCCCTCACCTCGCTC	*Nde*I
4211-R	CAT*AAGCTT*TCACCCCTCCAGCAGCCGCTGA	*Hind*III
C121G-F	GTCCGACCTGTTCGGCACCGGACACA	—
C121G-R	TGTGTCCGGTGCCGAACAGGTCGGAC	—
SLI_4212.P1	GCG*GAATTC*GGCGCCGACAGCGACAAGC	*Eco*RI
SLI_4212.P2	GCG*TCTAGA*GGCGATGGTCTGCGTCAAGGTG	*Xba*I
SLI_4212.P3	GCG*TCTAGA*GTGCGGACCTCCGACATCGAC	*Xba*I
SLI_4212.P4	GCG*AAGCTT*CTCCGTCGACTCGGTGGCC	*Hind*III
SLI_3189.P1	GATC*GAATTC*TGGGTGGGCACGAGCGTCTG	*Eco*RI
SLI_3189.P2	CATG*TCTAGA*TTGCTGCCGTACATCCAACC	*Xba*I
SLI_3189.P3	GATC*TCTAGA*CTGGCGCAAGGATAAGACAC	*Xba*I
SLI_3189.P4	CATG*AAGCTT*GTTCACCGGCAAGGAGAACG	*Hind*III
GlxA_F.NdeI	GATC*CATATG*AAAGACCGTGCCGGCCGC	*Nde*I
GlxA_R.BamHI	CATG*GGATCC*GAGCCACGGCCCGTTCATCCC	*Bam*HI
CslZ_F.NdeI	GATC*CATATG*TACGGCAGCAAGCCGGCCGGAAAC	*Nde*I
CslZ_R.BamHI	CATG*GGATCC*GTCGGTCTCCTTGGGCTGCTG	*Bam*HI
DtpA_F.NdeI	GATC*CATATG*CCCGACCAGTCCATTCC	*Nde*I
DtpA_R.BamHI	CATG*GGATCC*GCTGCCGAGCGAGGTGAGG	*Bam*HI
Ecuc_F.NdeI	GATC*CATATG*AGGCGGCTCGCGGAAGG	*Nde*I
Ecuc_R.BamHI	CATG*GGATCC*GGCGGCGTCGACCGAGAGTTC	*Bam*HI

### 2.4. Cloning and site-directed mutagenesis of *dtpA* and *glxA*

The SLI_4211 gene encoding DtpA was amplified from the genomic DNA of *S. lividans* strain 1326 by PCR using a forward primer (4211-F) with a flanking 5′-*Nde*I restriction site and a reverse primer (4211-R) with a flanking *Hind*III restriction site (table 3). The resulting PCR product (1134 bp) was ligated into the *Nde*I and *Hind*III sites of a pET28a (Kan^r^) vector (Novagen) to create an N-terminal His_6_-tagged construct (pET4211). The C121G variant of GlxA was created using a QuikChange mutagenesis approach using the C121G-F and C121G-R primers and the pET3188 vector as template (table 3).

### 2.5. Over-expression and purification of DtpA

pET4211 (Kan^r^) vector was transformed into *E. coli* BL21 (DE3) cells. Overnight precultures (low salt LB medium; Melford) were successively used to inoculate 1.4 l of high salt LB medium (10 g tryptone, 10 g sodium chloride, 5 g yeast extract per litre) with 50 mg ml^−1^ kanamycin and grown at 37°C, 180 r.p.m. At an OD_600_ of 1.2, 5-aminolevulinic acid (0.25 mM final concentration) and iron citrate (100 µM final concentration) were added consecutively for their use as a haem-precursor and iron supplement. Cultures were then induced by adding isopropyl β-d-thiogalactopyranoside (Melford) to a final concentration of 0.5 mM and carbon monoxide (CO) gas was bubbled through the culture for 20–30 s. Flasks were then sealed and incubated for a further 18 h at 30°C and 100 r.p.m. Cells were harvested via centrifugation (10 000*g*, 10 min, 4°C) and the cell pellet resuspended in 50 mM Tris/HCl, 500 mM NaCl (Fisher) and 20 mM imidazole (Sigma) at pH 8 (buffer A). The resuspended cell suspension was lysed using an EmulsiFlex-C5 cell disrupter (Avestin) followed by centrifugation (22 000*g*, 30 min, 4°C). The clarified supernatant was loaded onto a 5-ml nickel–nitrilotriacetic acid–sepharose column (GE Healthcare) equilibrated with buffer A and eluted by a linear imidazole gradient using buffer B (buffer A with 500 mM imidazole). The peak eluting at approximately 40% buffer B, found to contain DtpA, was collected, pooled and concentrated using a Centricon (VivaSpin) with a 10 kDa cut-off at 4°C and loaded onto a PD-10 desalting column to remove bound CO and imidazole followed by application to an S200 Sephadex column (GE Healthcare) equilibrated with 20 mM NaPi, 100 mM NaCl, pH 7. A major peak eluted at approximately 77 ml consistent with a monomer species with fractions assessed by SDS–PAGE then concentrated and stored at −20°C. DtpA concentrations were determined by UV–visible (vis) spectroscopy (Varian Cary 60 UV–vis spectrophotometer) using an extinction coefficient (*ɛ*) at 280 nm of 37 470 M^−1^ cm^−1^.

### 2.6. Over-expression and purification of holo- and apo-GlxA proteins

Wild-type (wt) GlxA (residues 354–645) and the C121G variant were over-expressed in *E. coli* BL21 (DE3) using the pET3188 vector (table 2) and purified as previously reported [14]. For the C121G variant, no CuSO_4_ was added before and after cell lysis so as to produce the apo-form of the variant. The production of apo wt GlxA required the adoption of an autoinduction procedure [40] using the medium 8ZY-4LAC-SUC [41]. All plastic and glassware was soaked in 0.1 M EDTA and rinsed extensively with doubly deionized water prior to use to ensure as metal-free conditions as possible. LB precultures were used to inoculate 400 ml 8ZY-4LAC-SUC cultures in 2 l baffled flasks with shaking at 180 r.p.m. 25°C for 48 h. Cells were then harvested and apo-GlxA was purified as previously described [14]. GlxA protein concentrations were determined using an *ɛ*_280_ of 78 730 M^−1^ cm^−1^.

### 2.7. Complex object parametric analyser and sorter measurements

Particle analyses using the complex object parametric analyser and sorter (COPAS) Plus profiler were performed as described previously [13,42]. Briefly, pellets were fixed with 4% formaldehyde for 30 min on ice, washed twice with phosphate-buffered saline, and stored at −20°C until further use. Samples were analysed using a COPAS Plus profiler equipped with a 1-mm nozzle (Union Biometrica, Holliston, MA). Pellets pass the laser beam over their longitudinal axis and data will be collected on the extinction (EXT) and time of flight (TOF) for all objects with a minimum TOF of 40. All experiments were performed in triplicate, and at least 2500 pellets were analysed per sample. The mean TOF values of the mutant strains were compared with that of the wt strain, which was set to 100%.

### 2.8. Western blot analyses

Mycelium was harvested from either TSBS liquid-grown cultures after 24 h of growth, or from solid R5 agar plates that had been overlaid with Track Edge membranes (Millipore) after 2 days of growth. The mycelia were washed with 10 mM Tris/HCl pH 7 buffer, and resuspended in 300 µl of the same buffer, followed by sonication on ice using a Bioruptor Plus (Diagenode). Complete lysis was checked by microscope, after which the lysed mycelium was separated into a supernatant and pellet fraction by centrifugation at 16 000*g* (4°C). Bradford analysis was used to determine the protein concentrations in the supernatant fraction, and 10 µg of protein was used for separation by SDS–PAGE on precast 7.5% mini-protean^®^ TGX Gels (BioRad) at 100 V for approximately 2 h. Proteins were transferred to polyvinylidene difluoride (PVDF) membranes (GE Healthcare) and incubated overnight with GlxA polyclonal antibodies (1 : 10 000) [20], which shortly before incubation mixed with EF-Tu1 antibodies (1 : 5000) [43] for analyses of extracts derived from liquid-grown cultures. Following 1 h of incubation with goat anti-rabbit alkaline phosphatase, detection was carried out with NBT/BCIP.

For the detection of recombinant GlxA proteins obtained from *E. coli,* 5 ng protein was used, and PVDF membranes were treated with diluted GlxA antibodies (1 : 20 000).

### 2.9. UV–vis spectroscopy and coupled peroxidase assay

Static UV–vis spectra were recorded using a Cary 60 spectrophotometer (Agilent). Hydrogen peroxide (H_2_O_2_) (Sigma) was prepared as required with concentrations determined using an *ɛ*_240_ of 43.6 M^−1^ cm^−1^. Resting state DtpA (Fe^III^) was prepared in 20 mM NaPi pH 7, 100 mM NaCl to a concentration of 10 µM in a 1 ml quartz cuvette (Hellma). Samples for UV–vis spectral analysis were prepared upon additions to the resting state DtpA of: 1 equivalent of H_2_O_2_; 1 equivalent of H_2_O_2_ followed by addition of 0.2 M glycolaldehyde (Sigma); 20 µM GlxA and 0.2 M glycolaldehyde; 0.2 M glycolaldehyde; 1 equivalent of H_2_O_2_ followed by 1 equivalent of ferrocyanide ([Fe(CN)_6_]^4−^). Catalytic turnover was measured using a coupled assay whereby production of H_2_O_2_ by GlxA in the presence of the substrates glycolaldehyde (0.2 M), d-galactose (0.6 M), d-glucose (0.6 M) and *N*-acetyl-d-glucosamine (0.1 M) (all from Sigma) was tested for in the presence of DtpA or horse radish peroxidase (HRP) and the subsequent oxidation of ABTS (2,2′-azino-bis(3-ethylbenzothiazoline-6-sulfonic acid) (Sigma). Samples were prepared in 1 ml cuvettes containing 20 mM NaPi, 100 mM NaCl, pH 7.0, 30 mM ABTS, 20 µΜ GlxA, 5 µM DtpA or 1 µl of HRP (10 mg ml^−1^) and the respective GlxA substrate, with reactions started by addition of GlxA. Oxidation of ABTS was monitored at 436 nm using a Hewlett-Packard 8453 diode-array spectrophotometer scanning between 190 and 1100 nm and thermostatted at 30°C. Turnover rate (*k*, s^−1^) was calculated from ((ΔA_436_/*ɛ*_ABTS_)/*t*)/[GlxA]), where ΔA_436_ is the absorbance change at 436 nm upon ABTS oxidation, *ɛ*_ABTS_ is the extinction coefficient of the ABTS cation radical oxidation product taken as 29.3 mM^−1^ cm^−1^, *t* is the time in s and [GlxA] is the total millimolar concentration of GlxA in the assay.

### 2.10. Tat-dependent secretion assay

Analysis of Tat-dependent protein secretion was performed as described [25,44]. PCR fragments encoding the candidate signal peptides were cloned as *Nde*I–*Bam*HI fragments into pTDW46, which contains the agarase gene lacking its original Tat signal sequence, expressed from the *dagA* promoter [44]. The empty plasmid pTDW46 was used as a negative control, whereas pTDW47 carrying a fragment for the original DagA signal peptide was used as a positive control. Candidate signal sequences were those from CslZ, DtpA, ECuC and GlxA (the putative signal peptide sequences are shown in table 3). All constructs were transformed to *S. lividans* TK24 and *S. lividans* TK24 *ΔtatC*. The agarase assay was performed by spotting each strain (1000 spores in a 10 µl drop) on MM-C medium, which contains agar as the sole carbon source [25]. After 5 days of growth, plates were overlaid with Lugol solution (Sigma), and staining was recorded by taking digital images after 45 min. Average diameters of clearing zones were calculated from 10 replicates per strain.

### 2.11. Bioinformatics

SLI database numbers refer to the genome of *S. lividans* 66 (alternatively known as 1326 [45]). To study the conservation of gene order, we used the synteny web service SyntTax [46]. Signal sequence predictions were carried out using PRED-TAT [47], and prediction of the Tat-motif and scores for the peptidase cleavage sites were obtained using TatP [48].

## 3. Results

### 3.1. Identification and characterization of copper-related morphogenes in *Streptomyces lividans*

**Figure 1.**
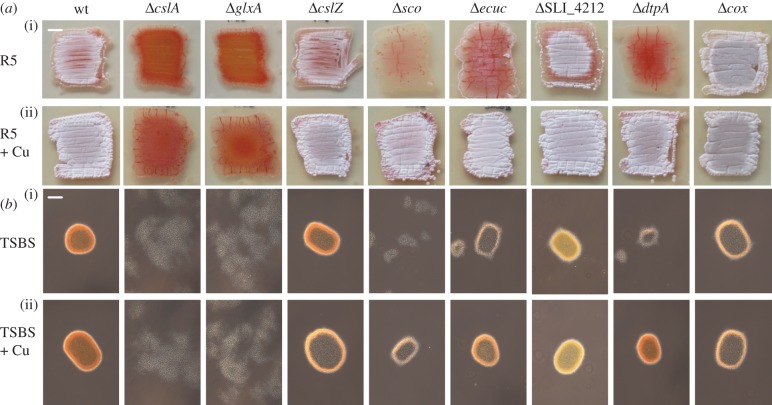
Phenotypic analysis of *S. lividans* strains lacking genes involved in Cu-related morphogenesis*.* The parental strain *S. lividans* 1326 is indicated as wt. (*a*) Morphology of strains after 3 days of growth on R5 medium in the absence (i) and presence (ii) of 10 µM CuSO_4_. Strains that have a white/grey appearance form reproductive aerial hyphae, while those that appear red-pigmented fail to do so and only form a vegetative mycelium. (*b*) Morphology of strains in TSBS medium after 24 h of growth in the absence (i) and presence (ii) of 10 µM CuSO_4_. Please note the difference between a pellet-forming strain (e.g. the wt strain) and a dispersed growing strain (e.g. the *cslA* mutant). Scale bar, (*a*) 500 µm and (*b*) 100 µm.

The *cslA* and *glxA* genes are conserved in streptomycetes and are organized in a larger gene cluster that also contains the *cslZ* gene (electronic supplementary material, figure S1). In *Frankia* species, this gene cluster is located adjacent to a *sco* homologue. Also in certain *Burkholderia* species, Gox-encoding genes co-occur with *sco* genes, suggestive of a direct functional correlation (electronic supplementary material, figure S1). In the genome of *S. lividans* 66 (also referred to as *S. lividans* 1326 [45]), *sco* is located elsewhere on the chromosome as a member of an operon containing the *ecuc*, SLI_4212 and *dtpA* genes (electronic supplementary material, figure S1). Given the putative correlation, deletion mutants lacking the majority of the coding sequences of either *cslZ,* SLI_4212 or *dtpA* were created as described in the Methods section, and compared with the previously generated mutants lacking *cslA, glxA, ecuc* or *sco* (figure 1). In line with earlier work, the *cslA* and *glxA* mutants failed to produce aerial hyphae on R5 agar plates, and development could not be restored to the mutants by the addition of 10 µM exogenous CuSO_4_ (figure 1). In contrast, the *cslZ* and SLI_4212 null mutants were identical to the parental strain and formed sporulating aerial hyphae after 3 days of growth. Deletion of *ecuc* led to a slight delay in aerial hyphae formation when compared with the parental strain (figure 1)*.* However, in contrast to *sco* mutants, the *ecuc* mutant progressed through development after 6 days, whereas no aerial hyphae were formed by the *sco* mutant (electronic supplementary material, figure S2). Notably, the addition of 10 µM CuSO_4_ to the medium rescued the morphological defects in the *ecuc* and *sco* mutants (figure 1 and electronic supplementary material, figure S2). The lack of development of these mutants is not a result of decreased CcO activity because the *cox* mutant, lacking CcO, develops normally (figure 1 and electronic supplementary material, figure S2 [23]). Interestingly, deletion of *dtpA*, encoding a putative haem peroxidase, also stalled development, which again could be restored by the addition of 10 µM CuSO_4_ to the medium (figure 1 and electronic supplementary material, figure S2) or by reintroduction of the gene (electronic supplementary material, figure S3). Development could not be restored to any of the mutants by the addition 10 µM of FeSO_4_, ZnSO_4_, MnSO_4_ or Co(NO_3_)_2_, showing that it is a specific effect mediated by the addition of Cu (electronic supplementary material, figure S4).

**Figure 2.**
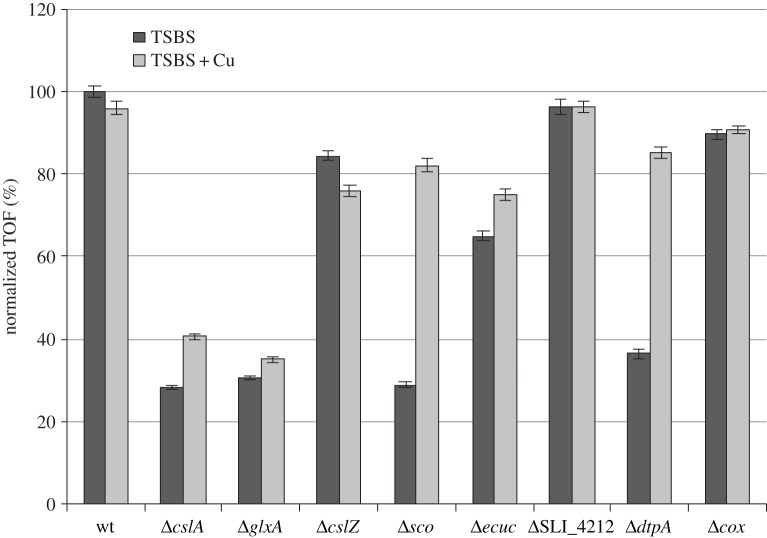
Average pellet sizes of *S. lividans* strains lacking Cu-related morphogenes. Strains were grown for 24 h in TSBS medium in the presence (light-grey bars) or absence (dark-grey bars) of 10 µM CuSO_4_. The average diameter of wt pellets obtained from TSBS medium was used as a reference and set to 100%. Error bars indicate 95% CIs of the mean.

Given the defects of *cslA* and *glxA* mutants in pellet formation, we analysed mycelial morphology of the other mutants lacking copper-related morphogenes both microscopically and quantitatively using a COPAS [13–15]. This revealed that the average size of mycelia of the *cslA* and *glxA* mutants decreased to 28% and 31%, respectively, of those of the parental strain, and this was not affected by the addition of 10 µM CuSO_4_ (figures 1*b* and 2). The *cslZ* and SLI_4212 mutants formed similarly sized pellets as the parental strain in both the presence and absence of additional Cu (figures 1*b* and 2). Interestingly, like in the *cslA* and *glxA* mutants, mycelia of the *sco* mutant were less dense and more open in TSBS-grown cultures (figure 1*b*). Their average size was reduced to 29% of the size of wt mycelia (figure 2). In contrast to *cslA* and *glxA* mutants, pellet formation was restored to *sco* mutants by extra Cu (figure 1*b* and 2). Deletion of *ecuc* had a relatively minor effect, with the average pellet size reduced to 65% of that of wt pellets, whereas pellet sizes slightly increased to 75% of wt values when Cu was added to the cultures (figures 1*b* and 2). Like in the absence of *cslA, glxA* or *sco*, mycelial pellets were also much smaller in *dtpA* mutants, yielding mycelia whose average size was reduced to 36% of the size of wt mycelia. In the presence of elevated levels of Cu, pellet morphology and size were similar to those of wt pellets (figures 1*b* and 2). Taken together, these data indicate that development and pellet morphology strongly depend on *cslA, glxA*, *sco* and *dtpA* and the bioavailability of Cu. Furthermore, it suggests an interdependence of the four proteins.

### 3.2. The absence of Sco, ECuC or DtpA affects GlxA maturation

**Figure 3.**
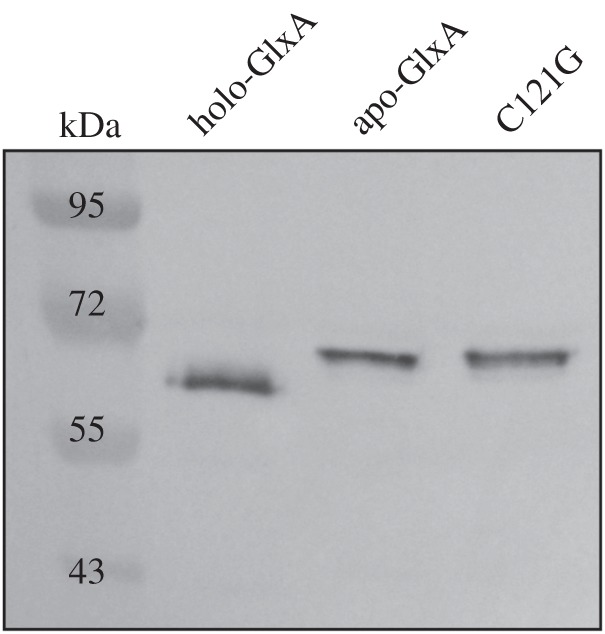
Immunoblot analysis of GlxA maturation from samples over-expressed in *E. coli*. GlxA is over-expressed and purified in either Cu-replete conditions (holo-GlxA) or Cu-starved conditions (apo-GlxA). Samples were prepared in SDS–PAGE buffer and heated at 95°C for 3 min. The C121G variant is unable to form the Tyr–Cys cross-link and migrates together with apo-GlxA, which requires the addition of Cu to form the cross-link. Molecular weight markers are indicated in kDa.

**Figure 4.**
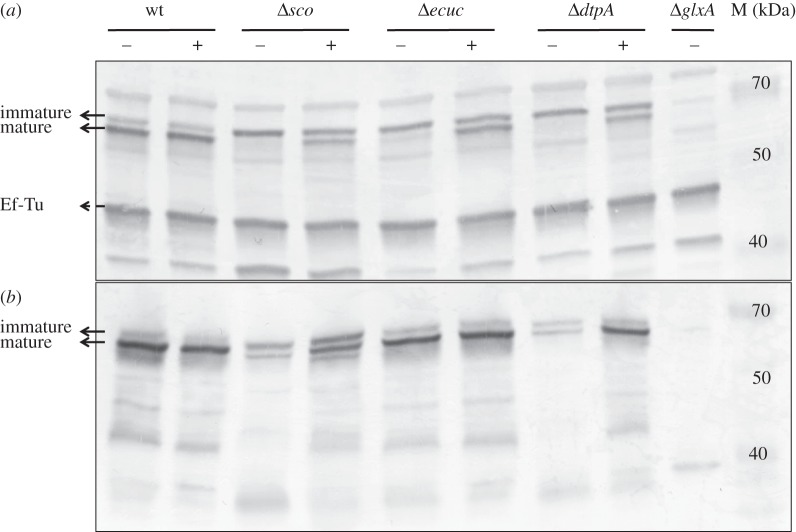
Immunoblot analysis of GlxA maturation in the wt strain and Δ*sco,* Δ*ecuc* and Δ*dtpA* mutants grown for 24 h in TSBS medium (*a*) or grown for 2 days on R5 medium (*b*) in the presence (+) or absence (−) of 10 µM CuSO_4_*.* The two bands indicated with the solid arrows represent GlxA, the upper band being immature GlxA (no Tyr–Cys cross-link) and the lower band being mature GlxA (with Tyr–Cys cross-link). The band indicated with a dashed arrow indicates EF-Tu, which serves as a control for the total amount of protein loaded on the gel. Molecular weight markers are indicated in kDa.

GlxA functionality requires the formation of a Tyr–Cys cross-link and the incorporation of a Cu ion [14]. Previous studies with fungal Gox have indicated that Cu is required to initiate the formation of the Tyr–Cys cross-link. This processed form migrates faster on an SDS–PAGE gel than the immature form (without the Tyr–Cys cross-link) [49]. To assess whether this maturation affects GlxA mobility, purified apo-, holo-GlxA and a C121G variant were run on an SDS–PAGE gel and migration patterns detected by GlxA polyclonal antibodies. From figure 3, it is apparent that apo-GlxA (prepared under Cu-starved conditions) and the C121G variant, in which the cross-linking Cys residue is replaced by a Gly, migrate slower than the holo-GlxA (prepared under Cu-replete conditions). This indicates that the Tyr–Cys cross-link absent in both the apo-GlxA and the C121G variant accounts for this retardation of electrophoretic mobility. Interestingly, both the mature and the immature forms of GlxA were detected in mycelia of *S. lividans* 1326 grown in TSBS cultures, with the mature form being more prominent (figure 4*a*). We then investigated whether the absence of Sco would influence the maturation of GlxA. In the absence of Sco, GlxA was exclusively found in its immature form, unlike in the parental strain (figure 4*a*). Interestingly, growth of the *sco* mutant in the presence of 10 µM CuSO_4_ led to the accumulation of the mature form of GlxA (figure 4*a*), consistent with the restored formation of pellets (see above). Given the changes in pellet morphology in the *ecuc* and *dtpA* mutant strains, we also verified the maturation pattern of GlxA in these mutants. In the *ecuc* mutant strain, only a small fraction of GlxA was in the mature form, with the majority being immature (figure 4*a*). However, no mature GlxA was identified in the *dtpA* null mutant. Like in the *sco* mutant*,* the mature form of GlxA reappeared in the *ecuc* and *dtpA* null mutants grown in TSBS supplemented with 10 µM CuSO_4_ (figure 4*a*). Deletion of *cslZ*, SLI_4212 or *cox* did not abolish GlxA maturation (electronic supplementary material, figure S5A). Altogether, these data implicate Sco, ECuC and DtpA in the GlxA maturation pathway and in pellet morphology under low levels of Cu in liquid-grown cultures.

We also analysed whether the developmental block on solid medium related to GlxA maturation defects. The amount of the mature form of GlxA was strongly decreased in the absence of Sco and DtpA, and could be increased by the addition of 10 µM CuSO_4_ to the medium (figure 4*b*). No major changes in the abundance of the mature form of GlxA were observed in the absence of ECuC, CslZ, SLI_4212 and CcO (electronic supplementary material, figure S5B).

### 3.3. DtpA acts as a peroxidase in the presence of GlxA

**Figure 5.**
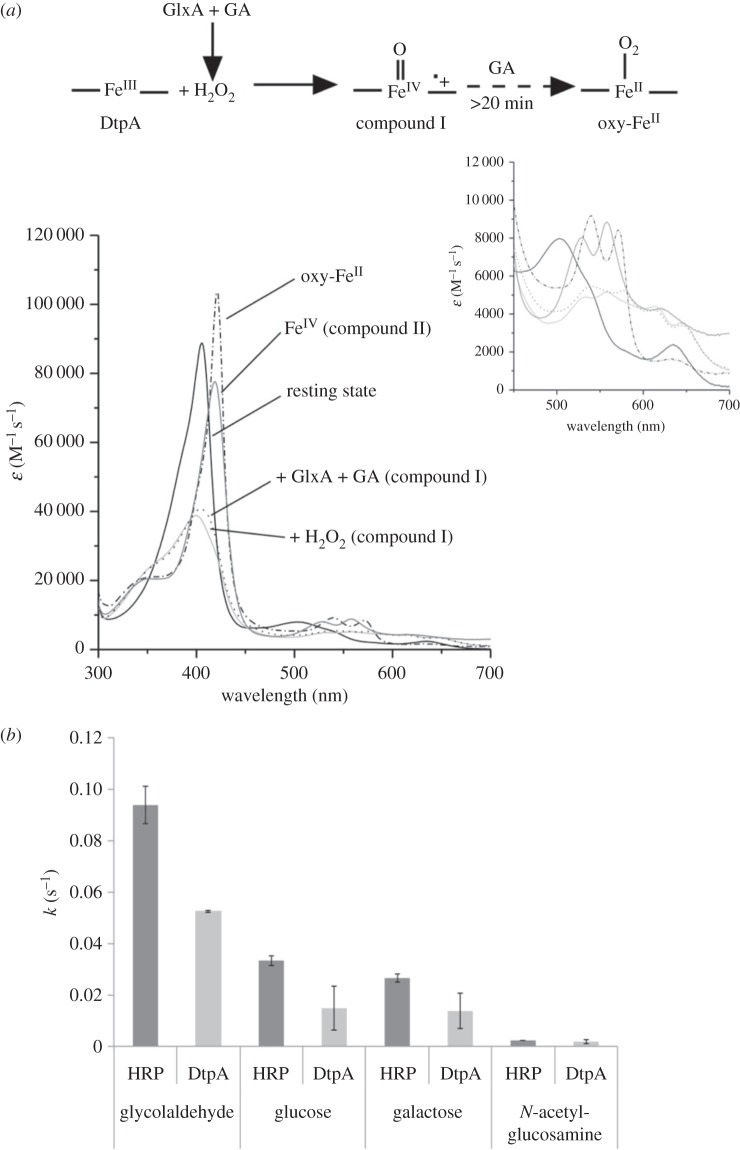
Peroxidase activity of DtpA. (*a*) Static UV–visible spectra of various haem oxidation states of DtpA (20 mM sodium phosphate pH 7, 100 mM NaCl) as illustrated by the reaction scheme. Addition of H_2_O_2_ (light-grey solid line), or addition of GlxA and glycolaldehyde (GA) (dotted line) to resting state (Fe^III^) DtpA leads to a compound I spectrum (formed within approx. 1 min). Over time (more than 20 min) the compound I species is converted to a species with an oxy-ferrous like spectrum (dashed-dotted line) in the presence of GA. Note that no compound II species is observed in this process. The compound II spectrum shown was generated by formation of DtpA compound I followed by addition of [Fe(CN)_6_]^4−^ (dark grey solid line). The inset shows a zoomed-in region of the weaker intensity absorbance bands. (*b*) Turnover rates (*k*) for GlxA with four different substrates (30°C) in the presence of HRP or DtpA determined through the subsequent oxidation of ABTS. Error bars indicate the standard deviation from triplicate experiments.

Purified DtpA gave a UV–vis spectrum with absorption maxima at 406 (soret band), 502, 635 nm and a shoulder at 540 nm, typical of a resting state ferric (Fe^III^) haem peroxidase [50–52] (figure 5*a*). Addition of H_2_O_2_ to resting state DtpA resulted in a shift to 399 nm and flattening of the soret band together with the formation of new absorption maxima at 530, 557, 614 and 644 nm. This indicates that DtpA had undergone a two-electron oxidation process to form compound I (haem• + Fe^IV^ =O), which over time and in the absence of a reducing substrate decays to the ferric resting state (*k* = 6.7 × 10^−3^ s^−1^ data not shown). This behaviour is typical of a peroxidase [50–52], which is consistent with the DyP-type peroxidase motif found in DtpA. On mixing equimolar amounts of DtpA and GlxA, no change in the DtpA absorbance spectrum was observed (data not shown). We have previously shown that under aerobic conditions GlxA is relatively inactive with substrates that are readily turned over by fungal Gox [14]. Upon addition of excess glycolaldehyde to the GlxA:DtpA sample (the best substrate for GlxA [14]), DtpA compound I is formed within a minute (figure 5*a*), providing direct proof that DtpA is a true peroxidase and GlxA is producing H_2_O_2_, which is subsequently used by DtpA. Over a period of time (greater than 20 min) compound I is transformed to a new species with absorption maxima at 420, 539, 571, 635 and 696 nm (figure 5*a*). This indicates that in the presence of glycolaldehyde compound II, i.e. a ferryl species (Fe^IV^ =O), which in the case of DtpA has absorption maxima at 419, 528, 557, 621 and 728 nm (figure 5*a*), is not formed. Instead, the new species has a spectrum that resembles an oxyferrous form (Fe^II^) suggesting that under the conditions employed DtpA eventually becomes fully reduced. The same spectral species is formed upon addition of excess glycolaldehyde to DtpA compound I generated through addition of one equivalent of H_2_O_2_, and on addition of excess glycolaldehyde to the resting state ferric (Fe^III^) enzyme (see electronic supplementary material, figure S6). This illustrates *in vitro* that glycolaldehyde is acting as a reductant and not interfering with compound I formation. Therefore, when GlxA is present, glycolaldehyde is its substrate leading to the production of H_2_O_2_.

One possibility that could arise is that GlxA activity is dependent on the type of peroxidase used. Prior to assessing whether GlxA activity could be affected in the presence of DtpA, native PAGE and analytical gel filtration chromatography were used to assess whether a physical interaction occurred. Neither method gave evidence to support a strong interaction between the two proteins (data not shown). To test whether GlxA activity is dependent on DtpA, a coupled peroxidase assay was performed comparing DtpA with horseradish peroxidase (HRP). Figure 5*b* compares the turnover rates (*k*, in s^−1^) for various substrates in the presence of HRP or DtpA. It is apparent that for all substrates tested no boost in activity occurs when DtpA replaces HRP.

### 3.4. CslZ and DtpA are exported via the Tat machinery

**Figure 6.**
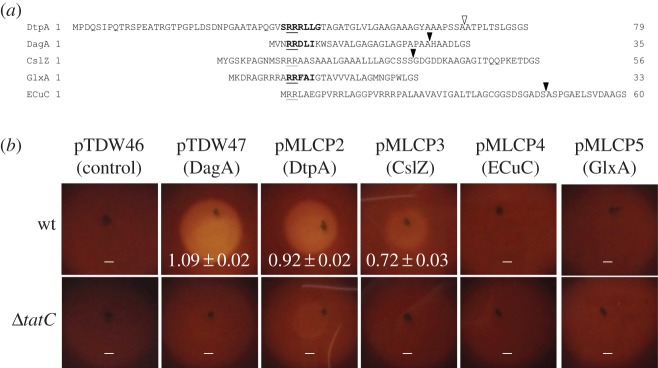
Tat-dependent protein secretion of DtpA and CslZ in *S. lividans*. (*a*) N-terminal signal sequences of the *S. lividans* DtpA, DagA, CslZ, GlxA and ECuC proteins. Boldface text highlights the twin-arginine motifs predicted by TatP, with the conserved arginines being underlined. The solid triangles at the C-termini indicate the predicted strong peptidase cleavage sites, while the open triangle indicates the predicted cleavage site in DtpA, which has a low cleavage site score. The amino acids Gly–Ser at the end of the signal sequences result from the introduced *Bam*HI restriction site used for their cloning. (*b*) Visualization of extracellular agarase activity after lugol staining of *S. lividans* strains grown on MM-C medium with agar as the sole carbon source. The used strains expressed the DagA protein without its signal sequence (pTDW46), or with signal sequences of DagA (pTDW47), DtpA (pMLCP2), CslZ (pMLCP3), ECuC (pMLCP4) of GlxA (pMLCP5). Halos are indicative for DagA secretion. No halos were observed when the constructs were introduced in the *tatC* mutant. Numbers indicate the mean diameter of clearing zones in cm with the corresponding standard error of the mean.

Proteins that are directed to the twin-arginine translocation (Tat) machineries have signal peptides with a canonical architecture. They have a relatively basic n-region at the N-terminus, which contains the highly conserved twin-arginine motif, followed by a hydrophobic h-region and a polar c-region with a signal peptidase recognition site [53]. Careful analysis of the signal sequence of DtpA indicates that it fulfils the criteria for being a Tat substrate (figure 6*a*). Indeed, the PRED-TAT tool for predicting Tat signals indicates with a reliability score of 0.998 that DtpA is exported via the Tat translocation channel. However, TatP predicts that the putative signal peptidase cleavage site of DtpA, after position 68 [SSA-AT], is weak with a score of 0.32, well below the cut-off of 0.51. PRED-TAT also indicates that other proteins encoded by the *cslA–glxA* locus or the *sco* operon could potentially be exported via the Tat secretion machinery, namely GlxA, CslZ and ECuC (figure 6*a*). To establish which of these proteins are true Tat substrates, we used a reporter system that makes use of secretion of the agarase protein DagA, which is strictly Tat-dependent [25,44]. If secreted, then DagA will degrade agar into its oligosaccharides, visible as a halo surrounding the colony after staining with iodine. Control colonies of *S. lividans* TK24 expressing DagA with its native N-terminal signal sequence were surrounded by a zone of clearing of 1.09 ± 0.02 cm. Zones of clearing were also observed when the N-terminal signal sequences of CslZ (0.72 ± 0.03 cm) or DtpA (0.92 ± 0.02 cm) were fused to DagA, consistent with the *in silico* prediction (figure 6*b*). In contrast, no halos were found with the putative Tat signal sequences of GlxA and ECuC (figure 6*b*). Furthermore, when DagA fused behind the signal sequences of DtpA or CslZ was expressed in the *tatC* null mutant of *S. lividans*, no zones of clearance were detected (figure 6*b*). Taken together, these results demonstrate that the N-termini of CslZ and DtpA are bona fide Tat signal sequences and that secretion of these two proteins depends on the Tat secretion pathway.

### 3.5. The addition of Cu restores GlxA maturation and morphogenesis to *tatA* and *tatC* mutants

**Figure 7.**
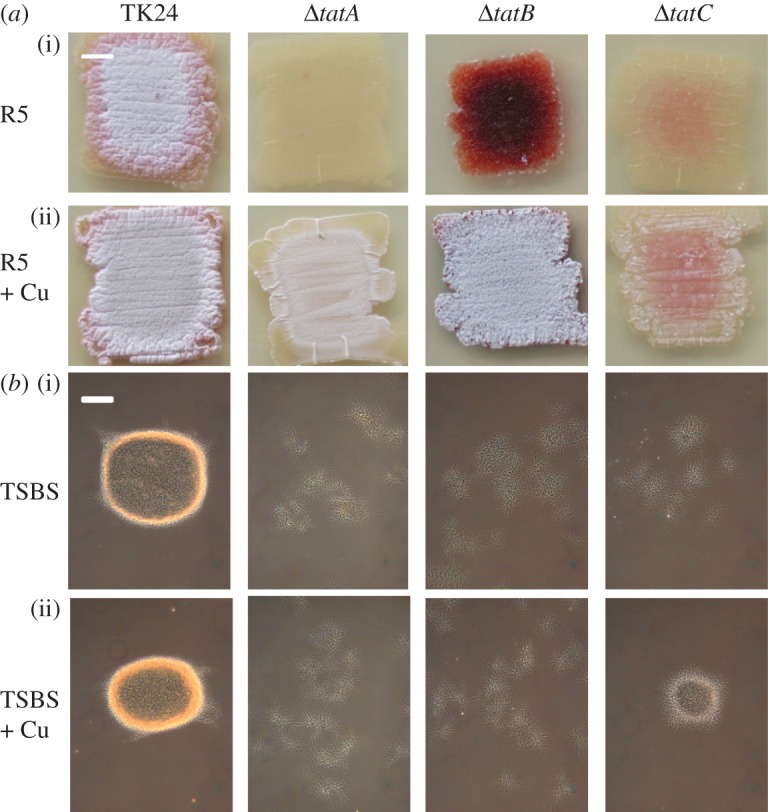
Phenotypic analysis of *S. lividans tat* mutant strains*.* The parental strain *S. lividans* TK24 is indicated as TK24. (*a*) Morphology of strains after 3 days of growth on R5 medium in the absence (i) and presence (ii) of 10 µM CuSO_4_. (*b*) Morphology of strains in TSBS medium after 24 h of growth in the absence (i) and presence (ii) of 10 µM CuSO_4_. Scale bar, (*a*) 500 µm and (*b*) 100 µm.

**Figure 8.**
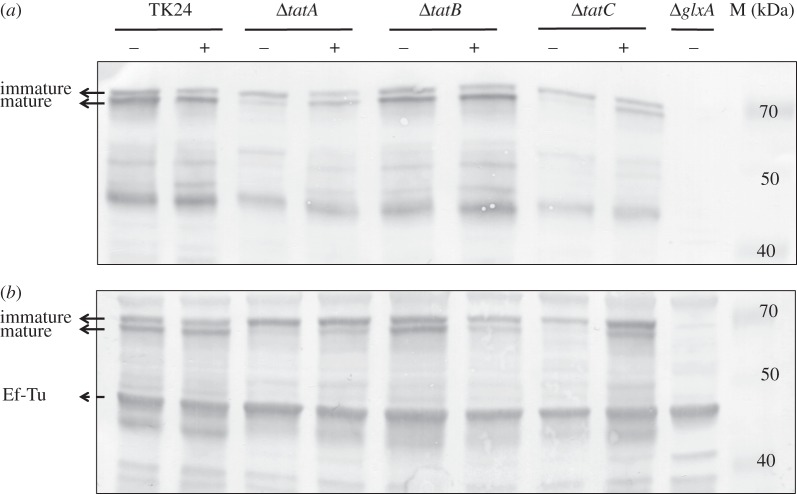
Immunoblot analysis of GlxA maturation in *S. lividans* TK24 and *tat* mutant strains grown for for 2 days on R5 medium (*a*) or grown for 24 h in TSBS medium (*b*) in the presence (+) or absence (−) of 10 µM CuSO_4_*.* The two bands indicated with the solid arrows represent GlxA, the upper band being immature GlxA (no Tyr–Cys cross-link) and the lower band being mature GlxA (with Tyr–Cys cross-link). The band indicated with a dashed arrow indicates EF-Tu, which serves as a control for the total amount of protein loaded on the gel. Molecular weight markers are indicated in kDa.

Considering that the Tat substrate DtpA influences development in a Cu-dependent manner, we speculated that the previously described morphological defects of *tat* mutants [32,33] might be restored by the addition of 10 µM CuSO_4_ (figure 7*a*). Surprisingly, this addition to R5 agar plates was sufficient to restore aerial growth in the *tatA, tatB* and *tatC* mutants. Addition of 10 µM ZnSO_4_, MnSO_4_ or Co(NO_3_)_2_ did not restore the formation of aerial hyphae when the strains were grown on R5 agar plates, whereas 10 µM FeSO_4_ only slightly improved aerial growth in the *tatB* mutant (electronic supplementary material, figure S7). Notably, Western blot analysis revealed that the addition of 10 µM CuSO_4_ led to an increase in the mature form of GlxA in the *tatA* and *tatC* mutants, similarly as observed for the *sco* and *dtpA* mutants on R5 agar plates (figures 4*b* and 8*a*). In contrast, mature GlxA was detected in the *tatB* mutant irrespective of the presence of 10 µM CuSO_4_.

We also analysed pellet formation and GlxA maturation in liquid-grown TSBS cultures in the presence or absence of additional Cu. As expected, the three *tat* mutants grew as dispersed mycelia in liquid-grown TSBS cultures without added Cu. As on R5 agar, the mature form of GlxA was present in the *tatB* mutant in TSBS-grown cultures without additional Cu, in contrast to the *tatA* and *tatC* mutants (figure 8*b*). The mature form of GlxA reappeared in the *tatA* and *tatC* mutants grown in the presence of 10 µM CuSO_4_, and, importantly, restored pellet formation to the *tatC* mutant (figure 7*b*).

## 4. Discussion

Morphological differentiation in streptomycetes is a complex process that depends on environmental conditions and extensive extracellular signalling between hyphae [1,11,54]. Over the last decades, a large number of so-called *bld* genes were identified, which are required for development and in particular on the reference media, namely R2YE (or R5) agar plates. In most cases, the precise function of these genes is not clear. Recently, it was demonstrated that several of the so-called ‘classical’ *bld* mutants are disturbed in desferrioxamine (DFO) biosynthesis [55]. DFO is a chelator that recruits iron (Fe) from the extracellular environment [56,57]. Development in some of these *bld* mutants, and notably *bldJ* and *bldK*, is restored by the addition of exogenous Fe to the culture, thereby bypassing the requirement for this chelator. Work from our and other groups has shown that in addition to Fe, Cu also plays a crucial role in morphogenesis [21,22,24,58]. The work described in this paper provides further molecular insights into the importance of significant levels of Cu for development, as we show here that many of the genes relating to what we have dubbed the Cu-trafficking pathway are required for aerial hyphae formation—and hence also for reproductive sporulation—when the bioavailability of Cu becomes limiting. A key member of this pathway is GlxA, which requires Cu for formation of a cross-linked Tyr–Cys cofactor and enzymatic activity [14]. We provide evidence that the novel *bld* gene *dtpA* encodes a Tat substrate that is involved in Cu-dependent morphological development. DtpA is required for GlxA maturation, together with the Cu chaperone Sco, and as a haem-containing peroxidase DtpA also provides an interesting link between the copper- and iron-dependent pathways leading to morphogenesis.

**Figure 9.**
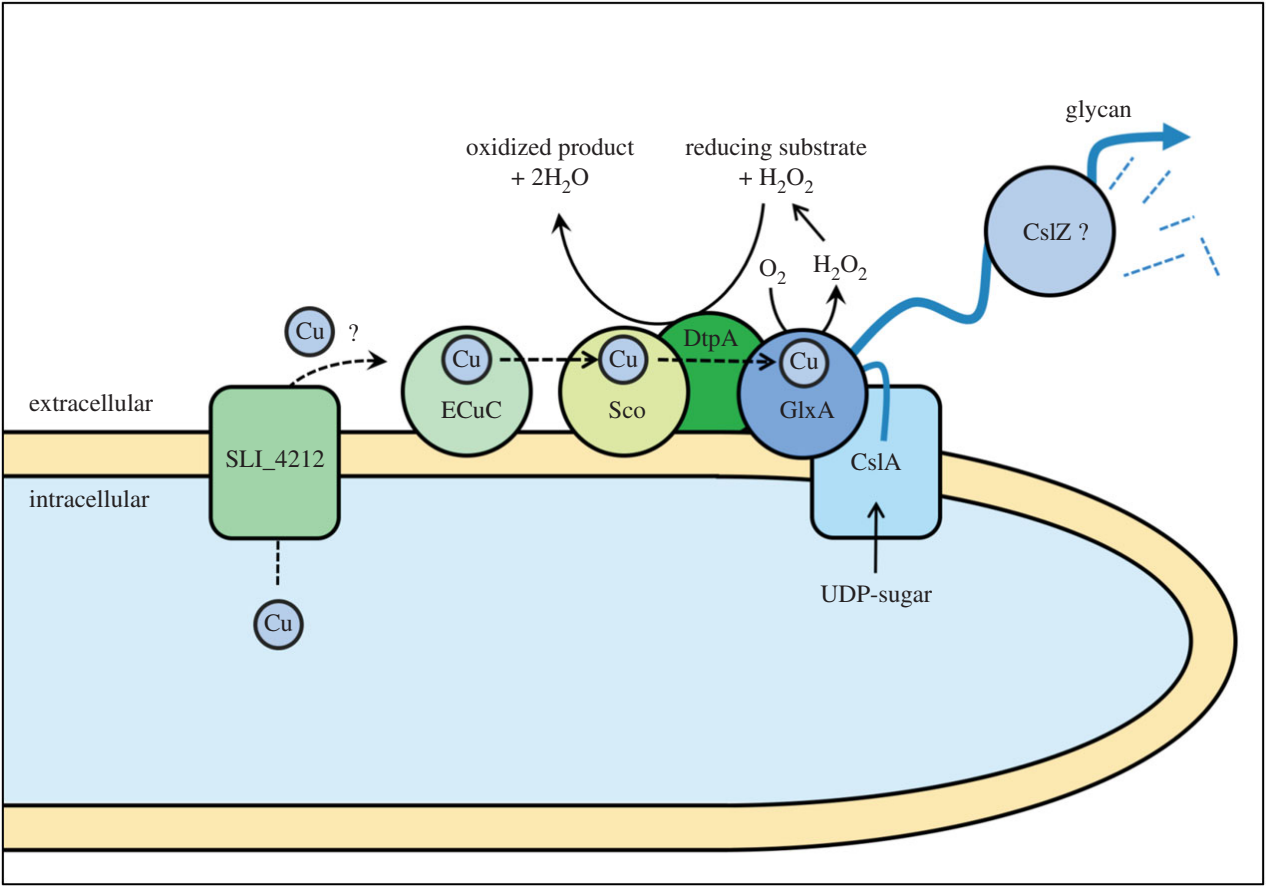
Proposed model for the Cu-dependent morphogenesis pathway in hyphal tips of *S. lividans*. Mature GlxA requires the incorporation of a Cu ion and the formation of a Tyr–Cys covalent bond. GlxA receives its Cu from the extracellular chaperone Sco, which in turn receives Cu from the lipoprotein ECuC. The putative Cu transporter SLI_4212 may be involved in shuttling Cu ions over the membrane, although its activity is not essential for GlxA function. DtpA is required for maturation of GlxA, possibly by changing Cu(I) to Cu(II) before transfer from Sco to GlxA. DtpA probably also removes the reactive H_2_O_2_, which is generated by GlxA while oxidizing its substrate. Mature GlxA acts cooperatively with the cellulose synthase like protein CslA in formation of an extracellular glycan, which may be processed by the endoglucanase CslZ.

The *dtpA* gene is located in a cluster of Cu-related genes that is present not only in streptomycetes but also in the remotely related actinomycetes *Frankia* sp. CcI3, *Thermobifida fusca*, *Nocardiopsis dassonvillei* and *Catenulispora acidiphila*. The clustering of *sco, ecuc* and *dtpA* in these organisms infers functional linkage between their gene products. Indeed, our work demonstrates that in streptomycetes these genes are all required for morphogenesis under conditions of Cu limitation, which is probably common in many laboratory media and in nature [59]. Based on past and current data, we propose a model for how the Cu chaperones Sco and ECuC function together with DtpA in the GlxA maturation pathway (figure 9). Eventually, mature GlxA functions together with CslA in the production and modification of an extracellular glycan that plays a crucial role in morphogenesis [14,15].

The absence of Sco has a dramatic effect on GlxA maturation, which can be compensated for by the addition of Cu to the medium. This connects well to our earlier work suggesting that Sco acts as the chaperone that provides Cu to GlxA [14]. Based on genomic context, this may also be true in other species, as previously suggested [60]. Sco in turn receives its Cu from ECuC [24], and our data indicate that the absence of *ecuc* also affects the correct maturation of GlxA, in particular under oxidizing conditions like in shaken liquid-grown cultures. However, the activity of ECuC is not essential for morphogenesis as the *ecuc* mutant formed a substantial aerial mycelium after prolonged incubation, and also formed pellets, albeit smaller, in liquid-grown cultures. This implies a role for ECuC in ensuring optimal Cu trafficking, but also indicates that Sco can obtain Cu in an ECuC-independent manner depending on the redox state of the environment. Something similar is true for GlxA, which in the absence of both Sco and ECuC can reach its mature conformation by the addition of Cu. How Cu is sequestered and transferred in the absence of these chaperones is not known and is under further investigation.

Our work clearly indicates that the maturation of GlxA depends on DtpA. We hypothesize that DtpA oxidizes Sco-bound Cu(I) to Cu(II). This would not only explain why GlxA maturation is impaired in both *dtpA* and *sco* mutants, but also why the CcO activity is reduced in the *dtpA* mutant ([26] and our unpublished data, 2015). Sco proteins bind both Cu(I) and Cu(II), and Cu transfer to acceptor proteins may depend on the oxidation state of the metal [60]. A role for DtpA in oxidizing metal ions would thus be very similar to the function of the *Bacillus subtilis* DyP-type peroxidase EfeB, which oxidizes Fe(II) to Fe(III) before uptake [61]. *S. lividans* also possesses an *efeB* homologue (SLI_2602), which is located in a gene cluster that contains genes encoding a lipoprotein (SLI_2601/*efeO*) and an iron transporter (SLI_2603/*efeU*). This organization is analogous to the *dtpA* gene cluster that contains genes for the lipoproteins Sco and ECuC and also for a metal transporter, in this case, the putative Cu transporter SLI_4212. Our data indicate that the *dtpA* gene cluster is tailored towards Cu trafficking, whereas the *efeB* gene cluster more likely influences iron homeostasis. Notably, the EfeO protein is reported to have a cupredoxin domain and to bind both Cu and Fe [62], again inferring cross-talk between both metals in pathways that are crucial for morphogenesis.

Our data demonstrate that GlxA produces H_2_O_2_ in the presence of glycolaldehyde, which is so far the best substrate determined for GlxA [14]. The H_2_O_2_ is then used by DtpA, thus contributing to protection of the hyphal tip from oxidative damage. Removal of the H_2_O_2_ leads to the formation of DtpA compound I. The conversion of compound I to compound II is not observed in our assay because of the reducing nature of excess glycolaldehyde leading to the slow formation of an oxyferrous species. However, compound II is detected in DtpA through the controlled reduction of compound I (as shown in figure 5*a*), indicating that DtpA is behaving as a true peroxidase and that the peroxidation mechanism is operable. Lack of a true substrate for GlxA therefore hampers a fuller investigation into the events occurring after compound I formation, but the formation of the latter clearly indicates a synergy between GlxA and DtpA. Cooperation between an oxidase and peroxidase has been demonstrated in some fungi, for instance between the glyoxal oxidase and manganese peroxidase in *Phanerochaete crassa* [63,64]. Therefore, the discovery of DtpA might help unravelling the substrate that is converted by GlxA, a crucial step towards discovering the composition and structure of the glycan produced by CslA and GlxA.

### 4.1. Can the Tat substrate DtpA explain the morphological defects of *tat* mutants?

The Tat secretion pathway is a major route for protein export in streptomycetes in comparison with most other bacteria [25,33], and resides at the hyphal tip [65]. *Streptomyces* mutants lacking *tatA, tatB* or *tatC* have morphological defects in liquid-grown environments, and also fail to develop a robust aerial mycelium [25,32–34]. Owing to the large number of predicted Tat substrates (between 145 and 189 [25,48,66,67]), no obvious candidates could be held responsible for these defects, which are undoubtedly caused by multiple missing proteins. However, our work shows that the Tat-secreted protein DtpA is a crucial substrate that, at least in part, explains some of the morphological defects observed in the *tat* mutants.

Adding Cu remarkably improved aerial mycelium formation by *S. lividans tat* mutants, which was also sufficient to restore the formation of pellets, albeit small, in liquid-grown cultures of *tatC* mutants. Western blot analysis indicated that the levels of mature GlxA in the *tatA* and *tatC* mutants were increased by the addition of Cu to the medium. These results are consistent with a model in which the TatA and TatC components of the Tat translocation machinery facilitate secretion of DtpA at the hyphal tip, where it contributes in the CslA–GlxA-dependent pathway of morphogenesis (figure 9). The addition of Cu also stimulated aerial growth in the *tatB* mutant, but this appears to be unrelated to the maturation status of GlxA. Given that in organisms that have a TatB protein it is also required for efficient Tat-mediated translocation [68–70], we suspect that, like in *tatA* and *tatC* mutants, DtpA is also not secreted in *tatB* mutants. However, besides its role in protein translocation, TatB may have an additional function, which is not shared with TatA and TatC. This would also be consistent with the observed differences in growth and morphology of the *tat* mutants (figure 7*a*). Such differential phenotypes were also observed for components of another important tripartite transport system, namely that of the PTS sugar transport system in *Streptomyces*, where mutants in *ptsH* have a phenotype that is distinct from that of *ptsI* and *crr* mutants [71]. Also, in this case, no additional function is known for any of these genes. We hypothesize that the developmental rescue of the *tatB* mutant by Cu is mediated by an unknown Cu protein that does not necessarily relate to the GlxA-dependent pathway described here. The observed variations in phenotype and GlxA maturation between the different *tat* mutants thus form an interesting starting point for further analysis of possibly specific roles of the individual Tat proteins in streptomycetes.

Our model suggests that the proteins involved in apical polymer synthesis may be organized in a larger complex. CslA is an integral membrane protein, whereas GlxA has an N-terminal membrane anchor and is shown to be membrane located [14]. Indeed, both proteins were shown to be tip-localized [15,17]. The chaperones Sco and ECuC are predicted lipoproteins [72], whereas DptA may also remain anchored to the membrane considering the presence of a transmembrane helix that overlaps with the Tat signal sequence. Given the weak signal peptidase recognition site, this offers the option that DtpA remains anchored to the membrane after transport, which is particularly important in liquid environments where the protein could otherwise diffuse away from its proposed functional site. Interestingly, the gene adjacent to *sco* encodes a copper-responsive protein with a so-called cohesin domain. Such domains are important for assembly of large macromolecular complexes, most notably the cellulosome of *Clostridium thermocellum* [73]. Whether this protein is involved in assembly of a large protein complex involved in hyphal tip glycan deposition is under current investigation.

## Supplementary Material

Supplementary Figures S1-S7

## Data accessibility

The datasets supporting this article have been uploaded as part of the electronic supplementary material.
