# Supplementary figures and images for: The DyP-type peroxidase DtpA is a Tat-substrate required for GlxA maturation and morphogenesis in Streptomyces
Source: Open Biol. 2016 Jan 6;6(1):150149. doi: 10.1098/rsob.150149 (PMC4736821; doi:10.1098/rsob.150149)

Figure S1

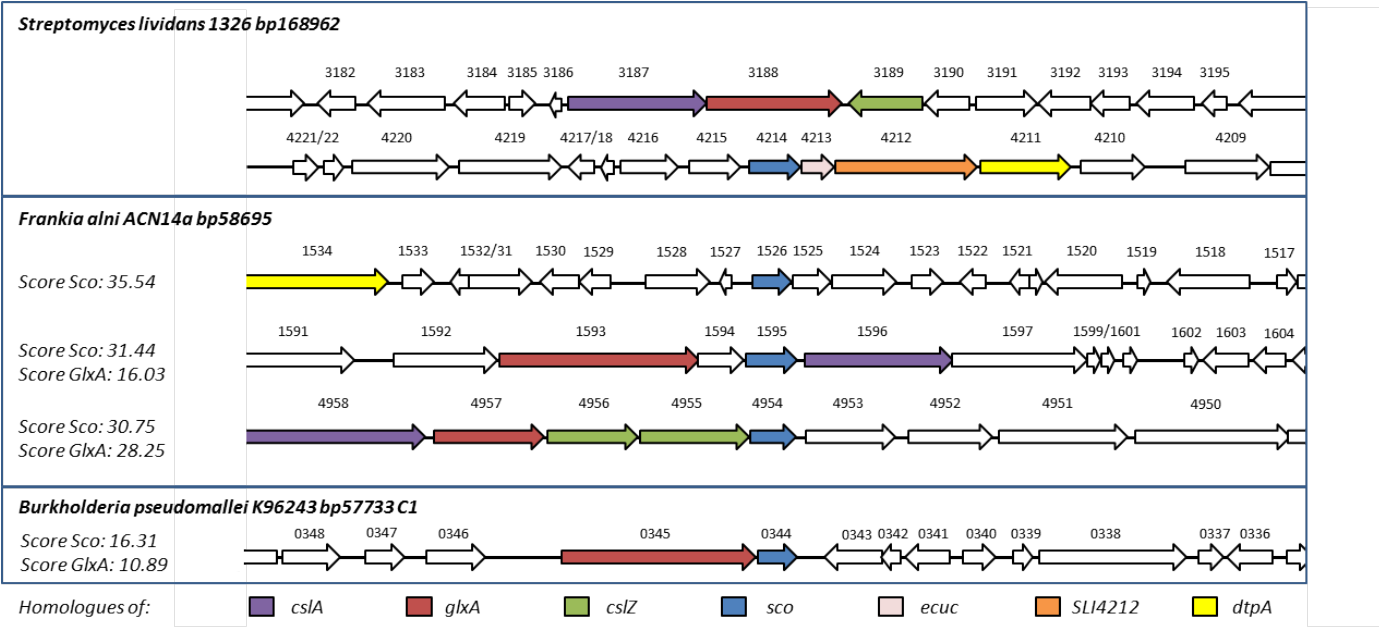

Figure S2

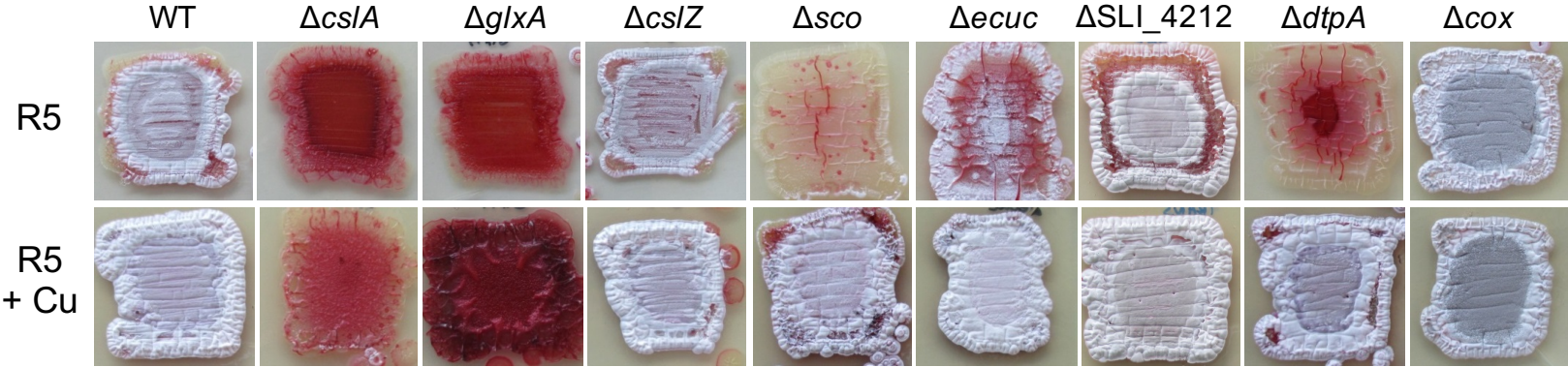

Figure S3

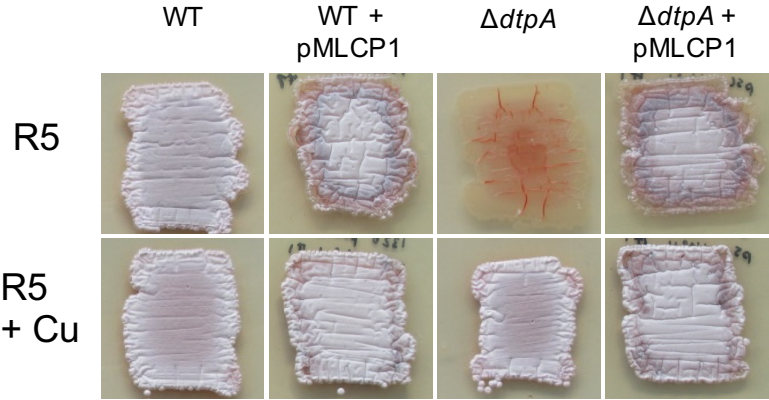

**Figure S4**

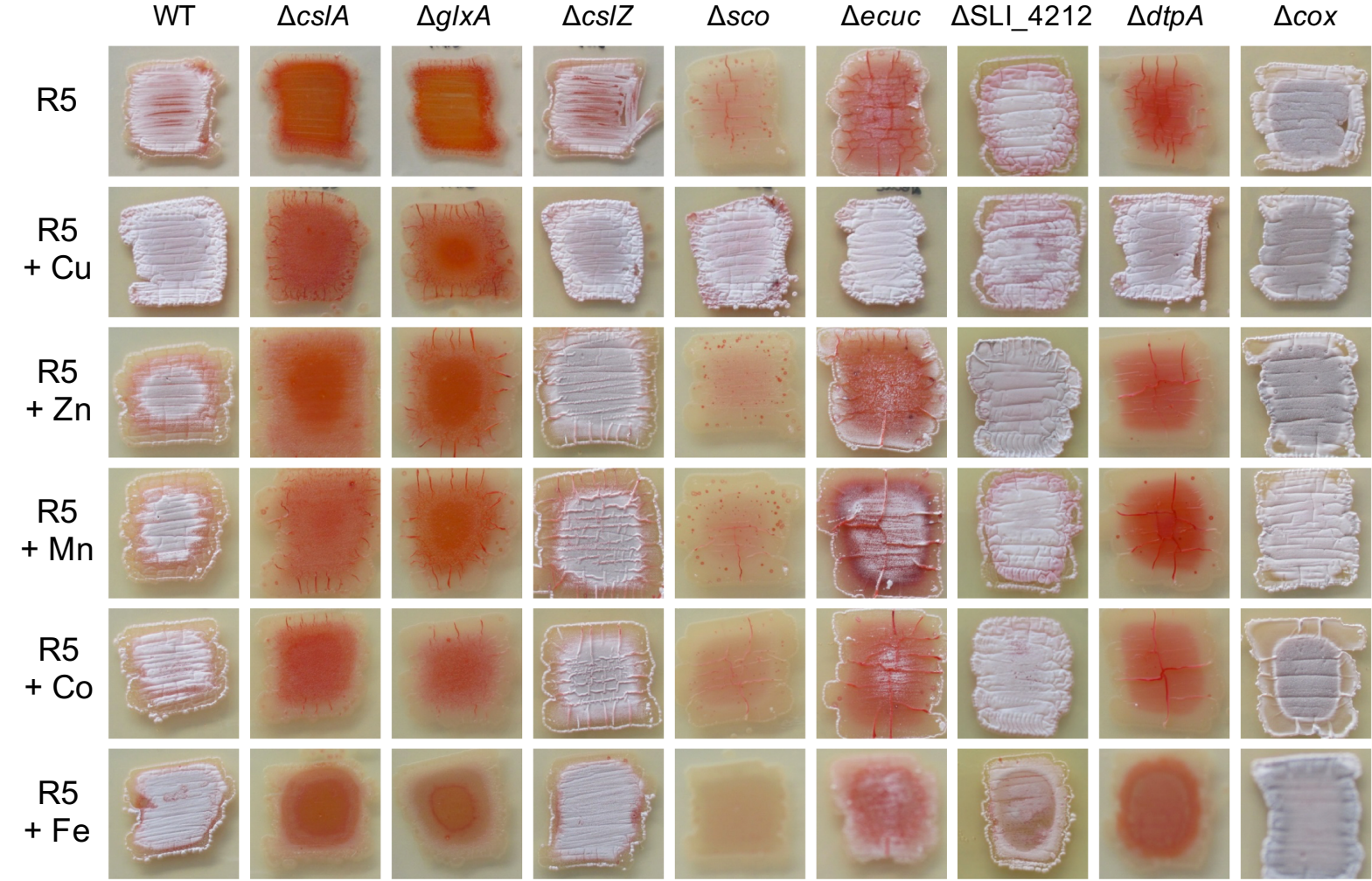

**Figure S5**

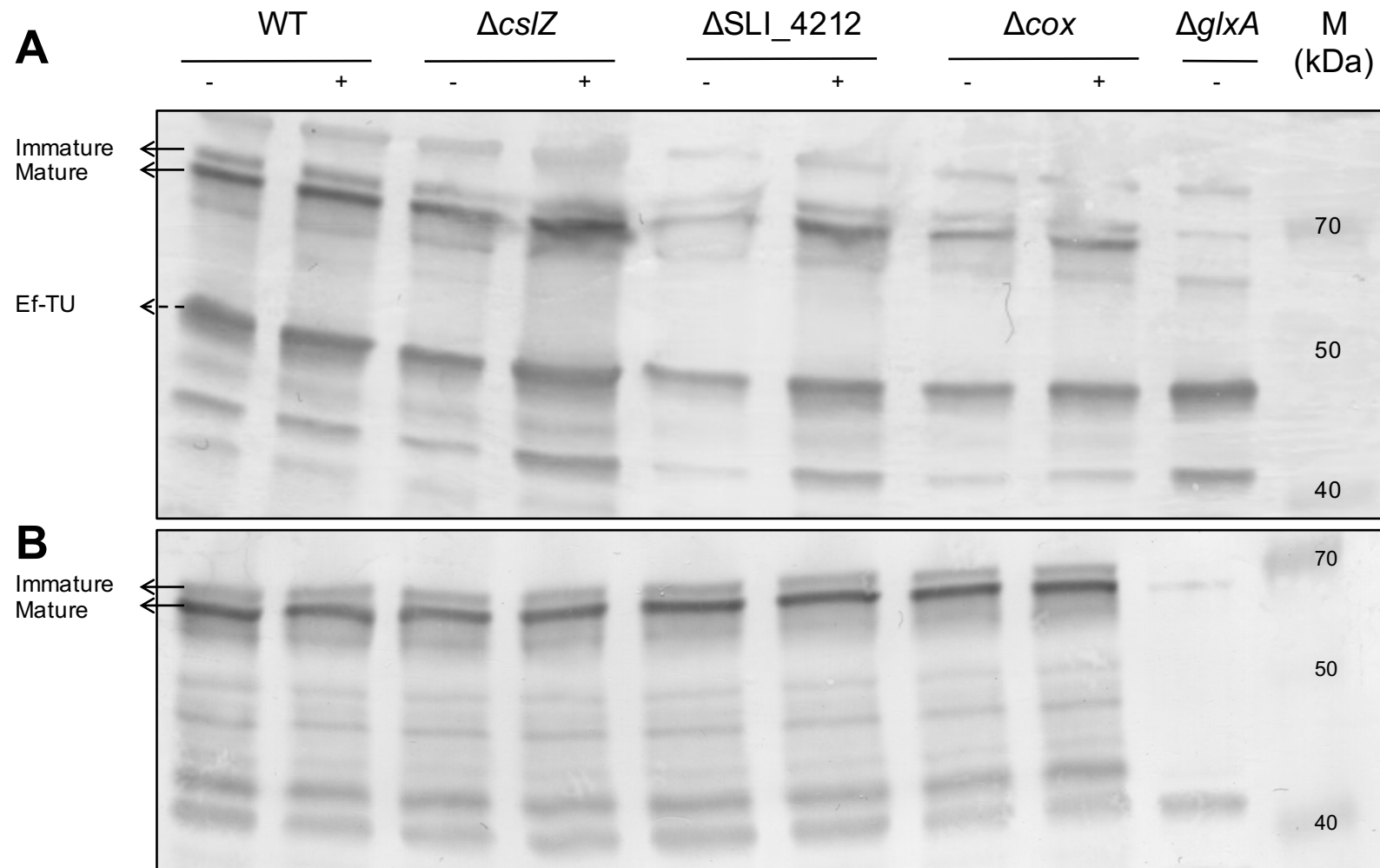

**Figure S6**

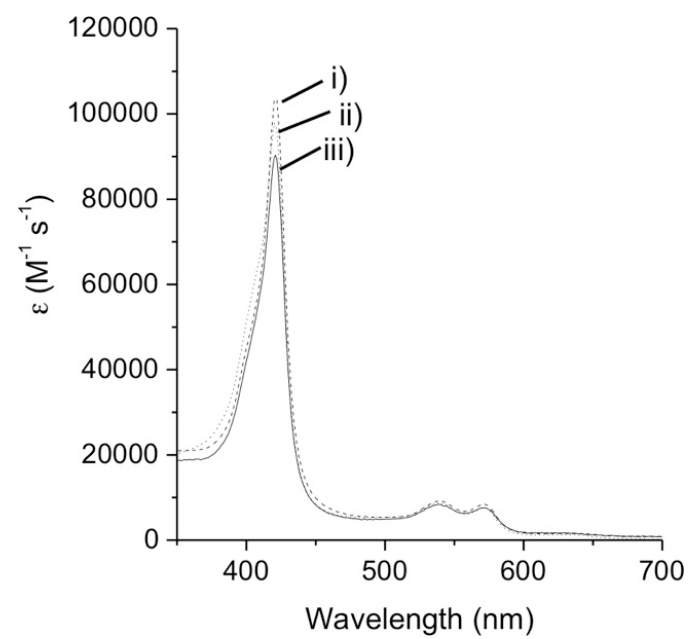

**Figure S7**

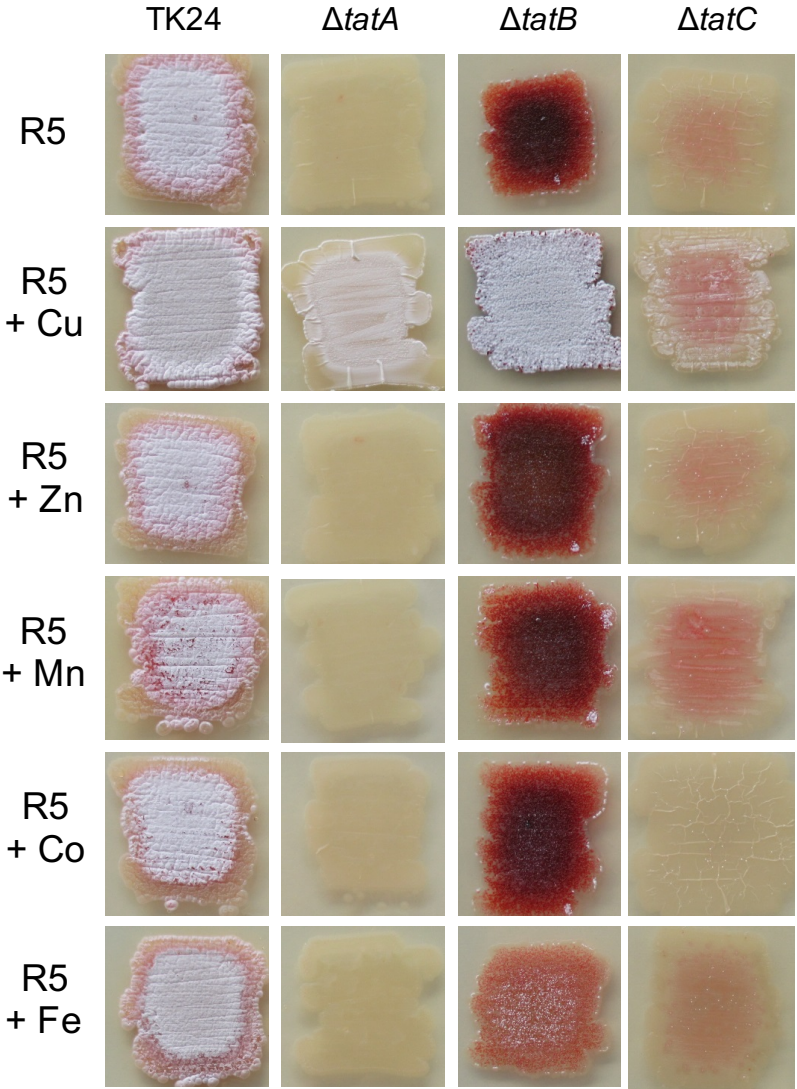

Supplement: Supplementary Figures S1-S7 [file rsob150149supp1.pdf]
